# Selecting effective incentive structures in health care: A decision framework to support health care purchasers in finding the right incentives to drive performance

**DOI:** 10.1186/1472-6963-8-66

**Published:** 2008-03-27

**Authors:** Thomas Custers, Jeremiah Hurley, Niek S Klazinga, Adalsteinn D Brown

**Affiliations:** 1 Department of Social Medicine, Academic Medical Center, University of Amsterdam, PO Box 22700, 1100 DE, Amsterdam, The Netherlands; 2 Health Results Team – Information Management/Ministry of Health and Long-Term Care, Toronto, Canada; 3 Department of Economics, McMaster University, Hamilton, Canada

## Abstract

**Background:**

The Ontario health care system is devolving planning and funding authority to community based organizations and moving from steering through rules and regulations to steering on performance. As part of this transformation, the Ontario Ministry of Health and Long-Term Care (MOHLTC) are interested in using incentives as a strategy to ensure alignment – that is, health service providers' goals are in accord with the goals of the health system. The objective of the study was to develop a decision framework to assist policymakers in choosing and designing effective incentive systems.

**Methods:**

The first part of the study was an extensive review of the literature to identify incentives models that are used in the various health care systems and their effectiveness. The second part was the development of policy principles to ensure that the used incentive models are congruent with the values of the Ontario health care system. The principles were developed by reviewing the Ontario policy documents and through discussions with policymakers. The validation of the principles and the suggested incentive models for use in Ontario took place at two meetings. The first meeting was with experts from the research and policy community, the second with senior policymakers from the MOHLTC. Based on the outcome of those two meetings, the researchers built a decision framework for incentives. The framework was send to the participants of both meetings and four additional experts for validation.

**Results:**

We identified several models that have proven, with a varying degree of evidence, to be effective in changing or enabling a health provider's performance. Overall, the literature suggests that there is no single best approach to create incentives yet and the ability of financial and non-financial incentives to achieve results depends on a number of contextual elements. After assessing the initial set of incentive models on their congruence with the four policy principles we defined nine incentive models to be appropriate for use in Ontario and potentially other health care systems that want to introduce incentives to improve performance. Subsequently, the models were incorporated in the resulting decision framework.

**Conclusion:**

The design of an incentive must reflect the values and goals of the health care system, be well matched to the performance objectives and reflect a range of contextual factors that can influence the effectiveness of even well-designed incentives. As a consequence, a single policy recommendation around incentives is inappropriate. The decision framework provides health care policymakers and purchasers with a tool to support the selection of an incentive model that is the most appropriate to improve the targeted performance.

## Background

The use of incentives in health care to improve performance is increasing rapidly worldwide. There are many reasons for this, but two consistent themes are the need to better align the incentives of providers with health system goals and the need to re-configure incentives to support new, devolved approaches to system governance. A continued misalignment between health service providers' compensation and key system goals drives the failure to deliver efficient and effective services for patient populations despite many efforts in the last decade to improve performance [1-3]. As many countries change governance principles of their health systems from rules and regulations toward devolved, results-driven systems that emphasize strategic planning and decision-making oriented towards performance [4,5], they have realized that success requires individuals and organizations in the system have an incentive to act on information and use their capacities to meet the heath system goals [6].

The health care system in the Canadian province of Ontario is an example of such system. The Ontario Ministry of Health and Long-Term Care is devolving planning and funding authority to community-based organizations, called Local Health Integration Networks (LHINs) while moving itself into the role of a steward. Incentives an integral part of the Ministry's efforts to ensure alignment across the Ontario health care system.

Using incentives to create alignment, however, is challenging because the link between incentives and the behaviour of individuals or organizations is not always straightforward. Evidence from a range of countries demonstrates that, at the macro level, the method of provider compensation influences practice patterns. A systematic review by Chaix-Couturie et al. shows that financial incentives have an impact on the use of health care resources, including: admission rates to, and length of stay in, hospitals; compliance with clinical practice guidelines; and achieving general immunisation rates [7]. A second systematic review of the impact of payment methods on the behaviour of primary care physicians found that those paid on fee-for-service basis provided more services than those paid by salary or by capitation [8]. At the micro level, however, the evidence is ambiguous. A systematic review of the impact of explicit financial incentives on quality of care yielded mixed results with respect to improving the processes and outcomes of care, access to care, and patient experience of care in a variety of populations and care settings [9]. Similar observations can be made with regard to public reporting as a tool to create alignment [10,11]. Consequently, although incentives can effect change, their effectiveness in eliciting the desired results depends critically on several (often poorly) understood aspects of the institutional environment in which they are used [12,13]. This poses a challenge for health care managers and purchasers who want to use incentives to improve performance.

Recognizing this, the Ontario Ministry of Health and Long-term Care developed a decision framework to assist policy makers and system managers in both the Ministry and the LHINs in designing effective incentive systems. Central to the framework is a series of questions that must be answered to identify what types of incentives can best achieve the desired performance improvement in a manner consistent with provincial values and principles.

This article presents the resulting framework which addresses fundamental questions that need to be considered in selecting an incentive model. Although the framework has been developed for the Ontario heath care system, the rationale behind its development and elements can be applied in other jurisdictions facing similar challenges. Development of the framework proceeded in three key stages: a review of the incentive models used in various health care systems and their effectiveness; development of principles to ensure congruence between incentive models and the values of the Ontario health care system; and finally, the development of a structured series of questions that form a decision-framework. A key contribution of the decision framework is its focus on offering explicit selection criteria that helps health care policy-makers and purchasers committed to using incentives identify the most appropriate incentive models to achieve the desired performance improvement.

## Methods

We identified incentive models and assessments of their effectiveness used in various health care systems through an extensive review of peer-reviewed literature on incentives in health care, the economic and organizational literature on incentives and organizational change (both empirical and theoretical) and other relevant publications. The literature search was conducted using a multidisciplinary database, ProQuest and a medical database, Medline, employing the following key words either alone and or in combination: "incentives", "motivation", "pay-for-performance", "organizational change", "performance management", "public reporting", "bonus", "return on investment and quality/performance", "funding/payment methods and quality/performance" and "quality and costs". We identified additional relevant material through the bibliographies of papers retrieved and by contacting experts about missing or unpublished papers. In addition, we searched the internet for information on incentives in health care applying the same keywords as used in the database searches as well as for additional background information on incentive initiatives mentioned in the articles identified through the literature search. Our inclusion criteria were English-language literature, and incentive literature published between 1 January 1995 and 1 May 2006, with the exception of the theoretical literature which we included regardless of publication date. In total we reviewed 85 articles and documents; we concluded our review once we felt that we had a comprehensive understanding of the literature and that further research would not lead to new insights.

The second phase was to select incentive models that seemed the most appropriate for the Ontario context based on a set of policy principles. First, an initial set of policy principles was developed by the primary researcher through an iterative process of reviewing Ontario policy documents and discussions with policymakers from the Ontario Ministry of Health and Long-Term Care (MOHLTC). Once the set of principles was articulated, an analysis was done on the congruence of the various incentive models with each of these policy principles. The decision as to whether a particular incentive model was congruent was influenced by both the number of principles with which the model may conflict and the political importance of any such policy principles. As a result, some incentive models that conflicted with one or more policy principles were selected while others that only conflicted with one policy principle were dismissed. The draft set of principles and selected incentive models were presented at two meetings. The first meeting, held in May 2006, included 17 participants representing policymakers from the ministry (10 participants), researchers (4 participants) and health service providers (3 participants). The results of this meeting then formed the basis for a second meeting in June 2006 with 13 senior policymakers from the MOHLTC. This meeting was part of a bi-weekly meeting cycle that dealt with developing a comprehensive policy framework for health system performance management in Ontario.

Following the two meetings, the analytic team led by the principal researcher applied the chosen principles and recommendations about the appropriateness of the various incentive models in building the draft decision framework. In the fall of 2006 the decision framework with the various incentive models was sent for comment and validation to participants of both meetings plus four additional experts in pay-for-performance, performance improvement and organizational change who had not participated in either meeting.

## Results

### Review of incentives, used in various health care systems, and their effectiveness

There is currently a wide range of new and established programs, particularly in the US, that employ financial and non-financial incentives to reward health service providers for achieving defined performance improvements [2,3,14-16]. A prominent example is the Centers for Medicare and Medicaid Services (CMS), Premier Inc. Hospital Quality Incentive Demonstration. Under this demonstration, that includes 268 hospitals, the highest-performing hospitals receive a higher reimbursement (bonuses) while the lowest-performing hospitals might be subject to withholds (penalties) based on their performance on a number of evidence-based quality measures for patients with heart attack, heart failure, pneumonia, coronary artery bypass graft (CABG), and hip and knee replacements [17]. In Europe, the English National Health Service has introduced several incentive programs, ranging from public recognition in the form of performance rating of trusts, increased autonomy for high performing trusts, and a bonus program for primary care practices [18,19]. In France, regional hospital agencies purchase hospital services and contract with public and private hospitals based on a range of structures, processes and outcome indicators [20]. In the Netherlands one of the largest health insurance companies has begun experimenting with performance-related bonuses and withdrawals in its contracts with hospitals, while another has introduced bonuses for general practitioners delivering excellent diabetes care. Australia has introduced a bonus program recognizing general practices that provide comprehensive, quality care, and that are either accredited or working towards accreditation [21]. Table 1 provides an overview of the incentive models used in health care that we identified in the literature. In addition to the distinction between financial and non-financial incentives, a further distinction can be made between direct and indirect incentives. Direct incentive models are intended to change the behavior of health service providers while indirect incentive models are intended to affect the behavior of health service providers through changes in the patient's choice of provider. We will briefly describe the most commonly used incentive models and their effectiveness in the remainder of this paragraph.

**Table 1:** Incentive models used in health care to change or enable an actors' behavior

	**Financial**	**Non-financial**
**Direct**	- Bonus - Performance based withhold - Performance-based fee schedule - Pay for activities - Shared savings contracts - Link regular payment (rate) increase to performance - Quality grants/performance fund - Financial award - Auto assignment	- Public reporting/recognition (*appeals to intrinsic motivation*) - Earned autonomy - Managerial replacement
**Indirect**	- Cost differentials for beneficiaries	- Public reporting/recognition (*appeals to patients who base their choice for a provider on quality*)

Despite the number of initiatives, good evidence regarding the effectiveness of these incentive programs is often lacking. Many of the evaluations suffer from weak designs that limit the ability to rule out other factors that may have contributed to the observed effects [22]. Generalizability is limited by the fact that the interventions typically occurred in a single location with unique characteristics. The published evidence regarding the most commonly used incentive models appears to yield mixed results (Table 2).

**Table 2:** Incentives – how effective are the most commonly used models

**Incentive Model**	**Lessons learned**
***1. Direct***	
**1.1 Financial incentive models**	
**Bonus**	Found some evidence that bonuses leads to performance improvement [24-29]. Bonuses are add-on money; provider can continue underperforming without negative consequences to the 'bottom-line'. Bonuses are not suitable for the long term: - funding with new money in times of raising health care costs not realistic. - funding by re-allocating existing funds across providers increases risk for access and equity.
**Performance based withhold**	Found limited evidence that it leads to performance improvement [60, 65]. However, non-performance leads to loss of income; as such it could be a stronger incentive than bonuses because on average, people place more value on losses than equivalent gains [37, 66]. Risks of creating resistance from providers which might lead to dysfunctional effects [67].
**Performance-based fee schedule**	Found very limited evidence that it leads to performance improvement [36]. Similar considerations as described under 'bonuses'.
**Pay for activities**	Found limited evidence that it leads to performance improvement [68]. There is a risk that it might freeze existing practices. There must be evidence that the activities that are subject to payment must lead to improved outcomes.
**Shared savings contract**	Found limited evidence that it leads to performance improvement [63]. Funding by anticipated savings as a result of the improvement is difficult because savings might be too small to generate a meaningful incentive, differences between anticipated and actual spending could vary significantly from year to year or some improvements don't result in savings.
**Link regular payment (rate) increase to performance**	Found limited evidence that it leads to performance improvement [61]. This might be partly explained by the fact that this method is relatively new. In principle it doesn't need additional money or reallocation of existing funds across providers although, they demand a higher reimbursement rate for putting their income at risk. The amount that is 'at risk' is relatively small, minimizing the risk of unintended consequences for patient care. Due to the relatively small amount of funding in question, resistance from providers may be minimal.
**Quality grants/Performance fund**	Found no evidence that it leads to performance improvement. It is often used as a tool to encourage innovation or to promote infrastructure investment and capacity building. Is not simple to administer. Resource intensive for health care purchaser (e.g. eligibility assessments and evaluation).
**Financial award**	Found no evidence that it leads to performance improvement. Used as a tool to stimulate innovation and superior performance. Because it is a one-time recognition, it doesn't overcome the misalignment caused by institutional arrangements.
**Auto assignment**	Has not been explored; from the beginning it was clear that this model is not feasible in Ontario.
**1.2 Non-financial incentive models**	
**Public reporting **(*appeals to intrinsic motivation*)	Found evidence that it leads to performance improvement; however, only for those performance aspects reported upon [69-71]. Concerns for their public image appear to be a key motivator for improvement. Although professional pride is a motivator, more concrete financial incentive could also be influenced by changes in hospital reputation like for example a hospital's ability to raise funds or recruiting and retaining qualified physicians and nurses [71]. Despite some doubts at the hospital management level in the NHS about the validity of the performance rating system they found it useful as a lever to influence staff behavior [56]. In general hospitals don't undertake many actions after reporting performance. In particular if the performance is enough in the eyes of a hospital [72]; poor performing hospitals will more frequently undertake activities [73].
**Earned autonomy**	No evidence found that it led to performance improvement. Stakeholders appreciate a greater degree of freedom but the effectiveness is highly dependent on [39]: - the nature of the freedom, which might lead to a conflict with a health care purchasers' effort for alignment. - the actual degree of freedom or reduced oversight, which is dependent on legislative requirements or other stakeholders like for example unions or professional bodies. - the commitment to award only those organization that perform well.
**Managerial replacement**	No evidence found.

***2. Indirect***	
**2.1 Financial incentive models**	
**Cost differentials for beneficiaries**	Has not been explored; from the beginning it was clear that this model is not feasible in Ontario.
**2.2 Non-financial incentive models**	
**Public reporting/recognition **(*appeals to patients who base their choice for a provider on quality*)	No evidence found that it led to performance improvement; patients don't find the performance information of hospitals very useful [10, 11].

Bonuses that reward providers with additional payments for achieving stipulated performance targets account for more than half of the current initiatives that link performance to payment, and are the most commonly used incentive model in the US [16,23]. An increasing number of studies suggest that such bonuses can improve performance in targeted areas [24-29]. The evidence is only suggestive, however, because for some these studies the effects are only partial [25] or there are limitations that makes it difficult to assess the true impact of the bonus program. These limitations are the lack of baseline including a control for pre-intervention trends in performance already occurring [26,30] or the lack of a concurrent control group [27] which means that the observed effects might be at least partially the result of other factors like increased monitoring. The evidence on the effectiveness of bonuses to drive performance improvement also includes studies indicating that bonuses had no effect at all [31,32]. The variation in effectiveness across settings may be due to a number of factors, including the small size of the bonus in some settings [9,33] or the inability of those targeted to affect the desired outcome [34]. While such bonuses can be effective, they are often not cost-effective: they must pay bonuses to practitioners who previously met the target in order to motivate the change at the margin among those who did not. In some cases, the majority of program resources go to those who already met the desired standard in the absence of the incentive. The quality incentives introduced by the NHS in 2004 provided additional payments of up to 25 percent of a practice's base income based on their performance with respect to 146 quality indicators related to clinical care for 10 chronic diseases, organization of care, and patient experience [35]. Although performance improved [26], the question that raised was whether the large financial investment – over 2 billion pounds – were an effective use of health care systems' resources.

The primary difference between performance-based fee schedules and bonuses is that the payment is ongoing rather than one-time or periodic [36].

An alternative, budget-neutral, incentive scheme places part of a providers' funding at risk based on the achievement of specified performance measures. Providers have to repay a portion of their payment to the health care purchaser if they fail to meet required performance levels (penalty), or the purchaser can set aside performance-related funding until a provider demonstrates that a standard has been meet. Prospect Theory, which describes how individuals evaluate potential losses and gains, suggests that people react to changes from the status quo rather than on final levels: individuals place more value on losses than equivalent gains [37]. This suggests that withholds or financial penalties may be more effective in driving performance improvement than bonuses. Under bonuses a provider is no worse off for not changing behaviour (they have simply forgone an extra payment), under withholds or penalties, a provider who fails to change is worse off: they must go through major changes just to maintain the status quo financially. The empirical evidence on the relative effectiveness of bonuses and penalties, however, is very limited. Negative incentives in the form of penalties or putting parts of the funding at risk based on performance are used less commonly than bonuses in existing programs among U.S. State Medicaid Programs [36], and their use is declining in new programs. Medicaid directors judge that withholds or penalties are detrimental to the operations of good incentive program because it creates ill will between the medical community and the state, which may result in decreases in provider participation [36]. Provider resistance also inhibits the use of withholds and penalties by health care purchasers and where used, avoiding the penalty requires a relative low level of performance improvement [13]. For example CMS agreed that there would be no penalties in the first two years and the 'penalty' threshold for the third year would be set as performance at or below the 10^th ^percentile of the performance in the baseline year.

A variation on withholds is to place some or all of regular funding increases at risk, so that future increases are linked to performance. This model might overcome some of the disadvantages of the withhold model and the bonus model. Unlike withholds it doesn't affect current funding levels which might reduce the resistance of health service providers and unlike bonus it doesn't require additional funding. Linking regular funding increases to performance would make over time the funding more performance oriented over time.

The remaining financial incentive models are 'performance funds', 'financial award' and 'quality grants' are used to stimulate innovation and to provide health service providers with the capacity to implement a quality-related program or research (see Table 2). Unlike quality grants and financial awards, performance funds are in general not competitive. Despite their common use in various health care systems, we were not able to find any evidence regarding the effectiveness of these three models.

Evidence for the effectiveness of non-financial incentive models such as public reporting and 'earned autonomy' are even more mixed. Public reporting of the performance of health service providers attempts to motivate performance improvement in two ways: by appealing to professional ethos and by harnessing market forces [38]. Although there is some evidence that public reporting can be a catalyst to improve performance, the effect is variable. The strength of the first pathway depends critically on the values of a provider, the leadership of an organization and the potential indirect consequences of being a poor performer as described in Table 2. However, intrinsic performance driven by values or leadership can particularly come under pressure if the economic incentives conflict with the values. The intended discipline of market forces is often weak because generally, patients don't use performance information in selecting a provider or do not have choice of providers because of capacity constraints [11]. A recent US study suggests that public reporting can be more effective when used in concert with financial incentives [38]. Hospitals subject to both public reporting and financial incentives improved quality more than hospitals engaged only in public reporting [38].

Even less is known about the effectiveness of "earned autonomy". Earned autonomy was introduced in the English NHS to create incentive for hospital trusts to meet national performance targets [18,39]. Under the presumption that provider's value autonomy, the policy offers a number of freedoms that fall into three broad categories: financial freedoms, reduction of the degree of central monitoring and more involvement in policy making [18]. Like public reporting, the incentive provided by earned autonomy depends importantly on the motivation of hospital managers. Unfortunately, earned autonomy as currently designed in the NHS provides little incentive effect for senior managers [39]; in part because a lack of commitment by the Department of Health in awarding or withholding autonomy reduced the credibility of the policy. In addition, earned autonomy doesn't address the potential financial misalignment caused by a funding system. As a result, earned autonomy seems to be a less appropriate model as an incentive for high performance.

### Risks of using incentives

Incentive schemes, both financial and non-financial, always risk generating unintended consequences. The principal type of unintended consequence is 'gaming', where participants find ways to maximize measured results without actually accomplishing the desired objective [40]. In healthcare, evidence of gaming was found in England as a result of the introduction of an annual system of publishing 'star rating' for public health care organizations [41]. For example, to meet the target '*Time spent waiting in accident & emergency' (emergency room) *some accident & emergency departments required patients to wait in queues of ambulances outside until the hospital in question was confident that that patient could be seen within four hours [42]. Roski et al [43] examined the effect of bonus payments on identifying patients with tobacco-use disorders and providing tobacco use disorders and providing tobacco cessation advice in large multi-specialty group practices. The incentive was associated with an increased documentation of tobacco-use status but not the provision of advice to quit smoking. If incentives are based on outcomes that vary with disease severity and inadequate risk adjustment there is a risk that health service providers select patients on the basis of the likelihood of a positive outcome. Shen [44] examined the effect of performance-based contracting on access to care among the most severely ill patients in a group being treated for substance abuse. The study showed that fewer clients with the greatest severity were treated in outpatient programs with the implementation of performance-based contracting suggesting that adverse selection was occurring in response to the financial incentive.

A second major concern with incentive programs is known in the economic literature as the multitasking problem [40,45]. If the goal of the payer is multidimensional and not all dimensions lend themselves [40] to measurement, rewarding performance based on available measures will distort effort away from unmeasured objectives.

Finally, another risk of using incentives to drive performance in health care is the potential to undermine the intrinsic motivation of people.

Although the risk of unintended consequences always exists and difficult to predict, Smith [46] and Marshall [47] identified a number of ways in which potential unintended consequences may be reduced. Examples are ensuring that the targeted health service providers are involved in the development of the incentive scheme, making use of existing, independent benchmarks if possible, seeking expert (both local and independent) interpretation of the performance measures, continuing evaluation of incentive systems and a process of feedback, and auditing of data.

Overall, the literature review showed that the ability of incentives to achieve the desired goals is neither direct nor straightforward. Furthermore, individual and organizations are motivated by many factors internal and external to their environment [9,12,33,48,49]. The effectiveness of an incentive will depends on the presence or absence of several key elements and as such, requires careful design involving those to whom incentives are targeted and ongoing evaluation of the effectiveness of the incentive program and potential unintended consequences. These findings are supported by other studies [9,33].

### Congruence of incentive models with values of the Ontario health care system

It is essential that the incentive models used be congruent with the values of the health care system. This was certainly true for the Ontario Ministry of Health and Long-term Care, which oversees the provincial public health care system and whose work is therefore informed by a strong value orientation. As noted above, the principles used to identify congruent incentive models were drawn from policy documents of the Ministry of Health and Long-term Care and discussions with Ministry officials.

The following four principles were adopted: (1) be fiscally prudent; (2) be simple to administer; (3) support a culture of continuous improvement, innovation and mutual learning and; (4) improve equity in and access to quality of health care services across all LHINs and health service providers.

When the various incentive models were assessed according to these four principles, the following models were regarded as appropriate to encourage desired behaviour and performance in LHINs and LHIN providers (see Table 3 for a summary of the analysis): (1) bonus; (2) pay for activities; (3) enhanced payment; (4) funding (rate) increases linked to performance; (5) financial award; (6) gain sharing; (7) grant; (8) performance fund (9) financial awards and; (10) public reporting/recognition.

**Table 3:** Summary analysis

**Principle**	**Consequences for selecting incentives**	**Recommendation**
**(1) Be fiscally prudent**	Incentives should not lead to additional costs for the health care system (no new money).	Exclude funding of bonuses/enhanced payment through new money without added value or without future savings.
**(2) Be simple to administer**	Incentive should be easy to implement and ideally be executed within existing policies, regulation and legislation.	Exclude 'Flexible oversight/greater autonomy' as an incentive as its design and implementation are too complex or might conflict with existing legislation or regulations.
**(3) Improve equity in access to quality of health care services across Ontario**	Incentives should not lead to differences in access to quality of health care services; instead, if possible, strengthen equity.	Exclude withhold of existing funding based on performance. Exclude bonus/enhanced payment funding via reallocation of funding from low to high performers.
**(4) Support a culture of continues improvement, innovation and mutual learning**	Incentives should: - not lead to tensions between ministry – LHIN and LHIN – health service providers. - focus on learning and improving rather than blaming. - encourage the sharing of best practices across LHINS and LHIN providers.	Exclude withholding of existing funding based on performance. Design public reporting in such a way that it prevents a "shame and blame" culture.

Some of the incentive models were included in the list despite the fact that using them could harm one or more policy principles. For example, unless bonuses are funded through savings (which has its own limitations as shown in Table 2), they will either lead to an additional increase in health care costs or, if funded through reallocation, might lead to differences in access to high quality care. Both are in principle undesired outcomes for Ontario policymakers. This makes it at first instance an inappropriate model to reward high performance; in particular as a reward on an on-going basis. However, for single and short term performance initiatives, the principle of being 'fiscal prudent' might be softened. It is not uncommon for policymakers to address a political priority by spending additional funding for a limited period such as required for initiatives e.g. to reduce wait times or to implement system wide information technology.

The incentive model 'earned autonomy' was identified as not appropriate even though it is congruent with three of the four principles. The experience in the UK showed that it's not simple to administer 'earned autonomy' [39]. Second, there was an increased recognition among the policymakers that certain freedoms which they initially regarded as a potential reward were actually driving forces for performance and as such should be eligible for all providers regardless of performance. Finally, the evidence on the effectiveness of 'earned autonomy' in creating change was weak. As a result, it was determined that 'earned autonomy' was not an appropriate incentive model for Ontario

### Choosing an incentive model in a given context: a decision framework

**Figure 1:**
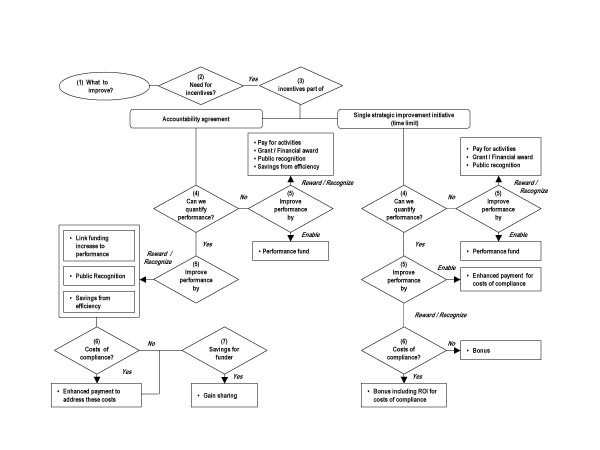
Decision framework for selecting incentives.

Choosing an incentive model and designing a specific incentive scheme are difficult tasks. The previous two steps led to a reduction of the number of acceptable incentive models that are used in various health care systems worldwide to nine models that have proven to be effective and that are congruent with the policy principles. The next step is to identify when each of these incentive models represents the best choice. We developed a decision framework to assist planners within both the Ministry and the LHINs in this task. The framework consists of series of related question set out in a decision tree (Figure 1). We consider these questions in turn and included an example related to diabetes care to illustrate the functioning of the decision tree.

The first question is to define the goals that a health care purchaser wants to accomplish through the incentives (what to improve?). An incentive scheme can't motivate health service providers to "do the right thing" if it is unclear what the right thing is. An example of a goal around diabetes care would be: 'providing comprehensive diabetes care in terms of appropriate and timely screening and treatment to reduce the risk of complications such as heart disease, blindness, and kidney disease'.

Having defined the key objectives, the second step is to ask whether there is a need for incentives. Poor performance can stem, in part or wholly, from a number of factors like the lack of knowledge, awareness, attitude or motivation of a health service provider, knowledge or patient attitudes, leadership, the attractiveness of an innovation or the economic and political context [50]. It is essential, therefore, to analyze why the system is currently under-performing and the potential role of incentives in improving performance. The need for introducing incentives or for altering existing incentives arises when current incentives reward undesired behaviour and fails to reward desired behaviour [51,52]. In our diabetes example, to provide comprehensive diabetes care, physicians need to perform activities such as for example Glycemic, blood pressure and lipid control, annual foot examination, annual tests of diabetic kidney disease, aspirin therapy for primary prevention in adults at increased risk for cardiovascular disease and smoking cessation counselling [53]. Neither fee-for-service nor capitation necessarily aligns incentives well (FFS may not compensate some of these activities well: capitation provides a general disincentive to provide services [8]). As a consequence, the lack of alignment makes incentives a potentially fruitful strategy to increase performance.

The third question in the decision-framework is whether incentives are part of an accountability agreement or used to promote the implementation of a single strategic improvement initiative. Accountability agreements generally outline, among other things, clear descriptions of responsibilities, objectives, performance expectations and measures and reporting requirements. Incentives can be tied to the performance expectations. In this context, the purpose of incentives would be to strengthen the accountability agreement. Incentive can also be linked to qualitative and quantitative goals of a single strategic improvement initiative with the aim of ensuring quick and broad implementation. The context in which incentives occur have implications for the choice of models. For example, the incentive model 'linking regular rate increases to performance' might, be difficult to realize for those improvement initiatives that are not part of an agreement as funding issues are subject to agreements. Bonuses might be appropriate for limited-term initial improvement initiatives but not to reward high performance on an on-going base. In our diabetes example we assume that the goal of providing comprehensive care is one of the performance expectations that are formulated in an accountability agreement.

The fourth question in the decision framework is the question whether the desired performance can be quantified. It is critical that the action or outcome tied to the incentive corresponds to the underlying performance objective. The usefulness of a performance measure in an incentive program will depend on its measurability and distortion [54]. An incentive can only be linked to what is measurable; it provides an incentive to do whatever is being measured. Measurability refers to a range of technical criteria that performance measures must pass [45]. Key among these are validity (does the measure capture what it is supposed to?), reliability (how reproducible are the measures when taken at different times or in different circumstances?), and responsiveness to change (will the measure discriminate between good and poor quality and be able to detect small but worthwhile improvements?). Distortion occurs when the performance measures incent a provider to take actions that are not congruent with the goals of the health care purchaser [16,55]. In many situations, the total value of an organization or health care system is not contractible, and thus cannot serve as the basis for an incentive contract [56]. For these reasons, most incentive contracts are based on measures other than the total value of the organization or health care system. The risk is that the marginal product of each type of action on the performance measure may differ from the marginal product of this action on the total value [55]. Some argue that when performance is unobservable (so there is a high risk of distortion) or the measures are technically not sound, it may be best to either reduce the incentive intensity to mitigate the risk that agents misdirect effort [57] or even to use no incentives for rewarding performance at all [45]. In our example, 'improving comprehensive diabetes care' can be measured through for example the indicators: A1C poor control > 9.0%, A1C control ≤ 7.0%, BP control < 140/90 mm Hg and LDL control < 130 mg/dL [58].

The fifth question in the decision framework is that of how incentives will improve performance. Health care purchasers can use incentives to enable or reward high performance. Enabling refers to the notion that high performance not only depends on the motivation but also on the ability to perform well. Rewarding performance reflects the common understanding on the role of incentives: to motivate high performance. Often, health service providers are motivated to perform well but are prevented from doing it by the current institutional arrangement. Being a high performer might increase costs for a provider [59]. For example, freeing up staff time, creating new positions like a director of quality and investing in education and information technology all increase costs in the short-term. The absence of a return on investment (ROI) on these costs or loss of income can form a barrier for pursuing the targeted performance improvement. In those cases, the focus of incentives is not on rewarding providers but to provide them with the necessary resources to become a high performer. The choice on how a health service provider can be compensated for its investments depends on the feasibility of information (see step four). If performance information is feasible, the investments can be compensated in both contexts (accountability agreement and single strategic initiative) by higher payments once the performance has improved. If performance information can't be quantified, the investments can be compensated with the help of a performance fund.

Incentives for rewarding and recognizing high performance goes one step further as it recognizes differences in performance by making a high performer better-off (either financially or non-financially) than an underperformer. A distinction can be made between recognizing and rewarding high performance. The purpose of recognition is to encourage organizations to put discretionary effort into the way they are functioning and to deliver superior performance. The difference with rewards is that recognition celebrates a one-time event while rewards are used to set direction and reinforce change [60]. The models that can be used to recognize high performance are: grants, financial awards or public recognition.

The choice on how to reward high performance will depend on two premises; first, as for enabling performance, on the ability to quantify performance and second, the context in which the performance expectation is formulated (see question 3). If the desired outcome is difficult to quantify, a health care purchaser can reward high performers by paying for activities (processes) that have proven to contribute to the desired outcome. Unlike outcome information, information on performed activities can often easily be obtained from administrative databases. If the desired outcome can be quantified, the selection of the reward will depend on the context. In the context of an accountability agreement the following incentive models combined are regarded as the most appropriate for rewarding high performers: (1) rate increase linked to enhanced performance; (2) public reporting and (3) savings from efficiency. 'Rate increase linked to enhanced performance' has two desirable features. First, similar as a withhold model, providers need to improve to maintain the status quo with regard to their bottom-line; however, unlike withholds, the risk of negative consequences for equity, access and resistance from health service providers might be lower as it on gradually makes the funding more performance oriented. Second, it doesn't require additional money and is therefore budget-conscious (although organizations might require a higher compensation for bearing the risk [45]). Public reporting strengthens the incentive to improve performance and more importantly, it increases accountability and educates the public about differences in health care. Finally, allowing organization to keep the surplus as a result of improved performance creates an incentive to improve efficiency which enables organizations to further improve care and develop innovative programs.

For incidental improvement projects or strategic priority projects with a short term, the use of bonuses is more appropriate in recognizing or rewarding accomplishments. Bonuses are easier to implement and generally do not require negotiations or rewriting existing contracts. Participation in a bonus program can also be established on a voluntary basis.

In the context of the diabetes care example it was determined that performance should be improved by rewarding high performance. As the performance expectation is part of an accountability agreement, the incentive is that providers will only receive the full rate increase when they improve their comprehensive diabetes care as measured by a set of indicators. The percentage of the rate that is at risk based on performance is subject to negotiation. In addition, their performance will be made public in comparison to other health service providers.

The sixth question addresses issues similar to those associated with enabling performance. The question is whether there are costs of compliance. Even if the purpose of the incentive is to reward high performance, the response of a provider to a reward will be influenced by their costs of performing the tasks necessary to improve. To be effective and to prevent undesired behaviour like gaming or tunnel vision, the reward needs to address these additional costs in its design. For example, the US-based Leapfrog Group helped payers determine how large a financial bonus is required to motivate hospitals to implement identified 3 patient safety practices. The work takes into account the hospital's cost of implementing the safety practice and the type of reimbursement structure (per-diem, case-rate, etc) [61]. In the context of an accountability agreement, the 'costs of compliance' can be compensated through higher payments; in the context of a single strategic improvement initiative, with feasible performance information, the costs of compliance need to be reflected in the size of the bonus.

In our example, improving diabetes care increase costs not well compensated by the current funding system, hence, the incentive will include enhanced payment (marginal costs) if performance targets are met.

The seventh question is whether there are savings for the health care purchaser. Various studies show that performance improvements can lead to savings for the purchaser or society [62]. In general health care purchasers can use the anticipated savings from the performance improvement as guidance on how big the bonus should be as is done for example by the Alliance in Wisconsin [63]. Using anticipated savings to reward high performers places great emphasis on those performance improvement initiatives that will lead to efficiencies and program savings. This makes it less appropriate as a source for rewarding increased patient centeredness or clinical quality. In some situations, the amount of savings might be too low to motivate change. Finally the savings can fluctuate which threatens the sustainability of the program. We believe therefore that the anticipated savings should be regarded in the context of an accountability agreement as a bonus in addition to the other more sustainable incentives already identified: rate increase linked to enhanced performance, public reporting and savings from efficiency. Instead of being the sole incentive, sharing the savings in the system strengthens existing incentives, and similar to a performance fund, provide organizations with additional resources to develop and implement new initiatives to improve their performance. In a non-contractual context, the savings can be used to guide the size of the bonus.

With regard to our example, studies have shown that improving diabetes care can lead to savings for the health care purchaser of up to 10–15% per patient per years. As a consequence, the health care purchaser is willing to share part of savings with those health providers who improved their comprehensive diabetes care.

### Implementation, evaluation and monitoring

In general, to generate behavioural change, incentives must be implemented together with tools and information such as education, support to facilitate the adoption of best practices and technical assistance. The health care purchaser needs to provide feedback to the health service providers on their performance and possible corrective action through dialogue between the parties [64].

The use of explicit incentives in health care is still quite recent, the collective knowledge base regarding their design and effectiveness is limited, and so their development remains largely a learning-by-doing process. This, together with the risk of unintended consequences, means that an incentive program needs to be monitored, evaluated, and improved on an on-going base. Two questions must be asked during the design phase to assure that the implementation of the program will support meaningful evaluation[13]: first, how can a health care purchaser tell if the incentive program is working; and what are potential unintended consequences. Demonstrating the effectiveness of an incentive program, requires a good research design, preferably with an appropriate control group, and good baseline data on the targeted quality measures. Only then can the main effect of the program can be evaluated in terms of change in performance compared either to unaffected population or the trend in performance prior to implementation [13]. To address the second question, health care purchasers should consider tracking a set of performance indicators that are outside of the incentive program [13].

The use of incentives requires ongoing adjustments to the program including modifying goals that lead to new measures and targets, adding or retiring measures once performance has topped out, adjusting targets for existing measures, and modifying the reward structure.

## Conclusion

Good performance depends on several factors, including the capacity to perform well, the availability of information and the motivation of an agent. The principle purpose of incentives is to motivate an agent (e.g. health service provider) to perform well when judged by the objectives of the principal (e.g. health care purchaser). This includes providing a provider with the incentive to develop the capacity to perform well, to acquire the right information, and to then act on it accordingly. Too often current systems of funding in health care fail to provide such incentives; indeed, it can directly frustrate the efforts of well-motivated health service providers trying to do the right thing, becoming itself a major barrier to good performance. The purpose of incentives in this article is therefore not only to motivate high performance by rewarding desired behaviour but also to provide health care providers with the capacity to leverage change.

Creating effective incentives is difficult. The design of the incentive must reflect the values and goals of the health care purchaser, be well matched to the performance objectives and reflect a range of contextual factors that can influence the effectiveness even well-designed incentives. Incentive design can be likened to building a house: forms follow function and the design of an incentive scheme depends on what you want from it.

The purpose of the decision framework presented in this paper is to assist health care purchasers in identifying the incentive model that is the most appropriate to improve the targeted performance. It is designed to prompt analysis and thought, not replace them. It can also support the communication between a health care purchaser and a health service provider. It opens the dialogue about what is expected, what is required from a provider's perspective and how a health care purchaser can provide these requirements to enable providers to deliver value. Key lessons from the literature on the use of incentives (both in health care and other sectors), are that no incentive model is appropriate for every context and that there is no single, best approach to create incentives for performance improvement. The decision framework therefore emphasizes fundamental questions that should be answered. It is flexible, tries to be balanced, reflects current knowledge, and is not meant to be applied mechanically as if it could automatically produce answers.

## Competing interests

The author(s) declare that they have no competing interests.

## Authors' contributions

TC: Conducted and summarized the literature review, conducted the preliminary analysis including developing the decision framework, drafted parts of the report which is the basis for this manuscript and drafted the manuscript;

JH: Worked close with TC on the literature review and the preliminary analysis including the development of the decision framework, read and validated the report and read and rewrote parts of this manuscript;

NK: Worked with TC on the design of the study, supported the preliminary analysis and the development the decision framework, drafted together with TC the report, read and provided detailed comments on this comments

AB: Initiated the study, participated in the preliminary analysis, the meetings with experts and policymakers and in the development of the decision framework, coordinated the study and read and validated the report and this manuscript

## Pre-publication history

The pre-publication history for this paper can be accessed here:


